# Potential Roles of the GRF Transcription Factors in Sorghum Internodes during Post-Reproductive Stages

**DOI:** 10.3390/plants13172352

**Published:** 2024-08-23

**Authors:** Min Tu, Zhuang Li, Yuanlin Zhu, Peng Wang, Hongbin Jia, Guoli Wang, Qin Zhou, Yuqing Hua, Lin Yang, Jiangrong Xiao, Guangsen Song, Yin Li

**Affiliations:** 1 Hubei Technical Engineering Research Center for Chemical Utilization and Engineering Development of Agricultural and Byproduct Resources, School of Chemical and Environmental Engineering, Wuhan Polytechnic University, Wuhan 430023, China; 2 School of Mathematics and Computer Science, Wuhan Polytechnic University, Wuhan 430023, China; 3 The Genetic Engineering International Cooperation Base of Chinese Ministry of Science and Technology, Key Laboratory of Molecular Biophysics of Chinese Ministry of Education, College of Life Science and Technology, Huazhong University of Science and Technology, Wuhan 430074, China

**Keywords:** GRF transcription factors, sorghum, gene expansion, RNA-seq, internode elongation, cell wall metabolism, co-expression analysis

## Abstract

Growth-regulating factor (GRF) is a plant-specific family of transcription factors crucial for meristem development and plant growth. Sorghum (*Sorghum bicolor* L. Moench) is a cereal species widely used for food, feed and fuel. While sorghum stems are important biomass components, the regulation of stem development and the carbohydrate composition of the stem tissues remain largely unknown. Here, we identified 11 SbGRF-encoding genes and found the *SbGRF* expansion driven by whole-genome duplication events. By comparative analyses of *GRF*s between rice and sorghum, we demonstrated the divergence of whole-genome duplication (WGD)-derived *OsGRF*s and *SbGRF*s. A comparison of *SbGRF*s’ expression profiles supports that the WGD-duplicated *OsGRFs* and *SbGRF*s experienced distinct evolutionary trajectories, possibly leading to diverged functions. RNA-seq analysis of the internode tissues identified several *SbGRF*s involved in internode elongation, maturation and cell wall metabolism. We constructed co-expression networks with the RNA-seq data of sorghum internodes. Network analysis discovered that *SbGRF1*, *5* and *7* could be involved in the down-regulation of the biosynthesis of cell wall components, while *SbGRF4*, *6*, *8* and *9* could be associated with the regulation of cell wall loosening, reassembly and/or starch biosynthesis. In summary, our genome-wide analysis of *SbGRF*s reveals the distinct evolutionary trajectories of WGD-derived *SbGRF* pairs. Importantly, expression analyses highlight previously unknown functions of several *SbGRF*s in internode elongation, maturation and the potential involvement in the metabolism of the cell wall and starch during post-anthesis stages.

## 1. Introduction

Growth-regulating factors (GRFs) are found to exit in a wide variety of land plants, playing pivotal roles in various aspects of plant development and growth regulation [1]. As a plant-specific transcript factor family, GRF has been identified as a key regulator in the development of the leaf, stem, flower, root and seeds [2,3,4,5,6], as well as in the coordination of the growth process under stress conditions, such as high salinity, drought, low temperature and so on [7,8,9,10,11].

In the context of organ development, GRFs have been shown to interact with other transcription factors and coactivators, such as GRF-interacting factors (GIFs), to modulate gene expression patterns that influence cell proliferation and expansion [12,13,14]. For instance, the *Arabidopsis thaliana GRF* family members *AtGRF1* to *AtGRF9* have been functionally characterized in leaf and cotyledon growth, with their overexpression leading to enlarged organs due to increased cell size rather than cell number [15]. AtGRF3 regulates leaf development and longevity by activating the expression of zinc finger homeodomain transcription factor *HB33* [16]. Further, some *GRF* members are targeted by the conserved miR396, thus comprising the miR396-GRF/GIF module to serve as a central post-transcriptional mechanism to regulate *GRF* expression levels, leaf and stem development and the coordination between stress responses and growth [2,12,16,17,18].

Functional studies have revealed that GRF proteins have two specific conserved domains (i.e., the QLQ and WRC domains) essential for their molecular functions [2,15,18]. The QLQ domain is accompanied by many aromatic/hydrophobic amino acids and functions as a protein–protein interaction domain, while the WRC domain is typically associated with DNA-binding ability and contains a putative nuclear localization signal [2,15,19]. Other reports documented several motifs of GRF proteins with putative functional relevance, including the TQL motif and the GGPL motif [20]. It has been established that the expression of GRFs could be induced under multiple hormonal treatments [21,22,23]. Various functions of GRFs in the model plant species (i.e., Arabidopsis and rice) have been extensively studied. GRFs control leaf growth and plasticity, promote grain filling, increase grain size and weight, and contribute to the response and adaptation to environmental stresses [24,25,26]. The conserved and divergent roles of GRFs and their regulation by the miR396 family have been systematically reviewed to highlight the potential values for yield improvement [25]. More recently, a comprehensive review summarized the functions of GRFs in controlling organ growth and plasticity and environmental responses. Interestingly, the molecular mechanisms regulating GRFs at multiple levels, including transcriptional and post-transcriptional controls, as well as miRNA396-mediated regulation and protein–protein interactions, have been thoroughly described, providing a molecular landscape to design modifications for biotechnological applications of GRFs [26].

Owing to the functional importance of the GRF family in plant growth and yield-related traits, this family has been genome-wide characterized in several Poaceae crops. Poaceae species are agriculturally important because they include several staple crops feeding billions globally. A total of 96 GRF-encoding genes have been identified in eight Poaceae crops, including *Brachypodium distachyon* L., *Hordeum vulgare* L., *Oryza. sativa* L. ssp. *indica*, *O. rufipogon*, *O. sativa* L. ssp. *japonica*, *Setaria italic*, *Sorghum bicolor* and *Zea mays* [27,28]. *SbGRF*s have also been genome-wide characterized [29]. While *GRF* genes were identified in the above-mentioned crops, functional studies were mainly performed on rice, emphasizing the importance of understanding the conservation and divergence between OsGRFs and those from other Poaceae species [25]. In rice (*Oryza sativa* L.), several GRFs have been functionally studied. For example, OsGRF1 is involved in gibberellic acid (GA)-induced stem elongation [19], while OsGRF7 affects plant architecture by regulating GA, auxin and cell length and arrangement [6]. The rice *grain length and weight 2* (*GLW2*) locus encodes OsGRF4, and the mutation of *OsGRF4* perturbs the cleavage site of miR396c and causes alleviated expression levels of *OsGRF4*, leading to a larger grain size and enhanced yield [30]. Similar to the well-known functions of AtGRFs, the miR396-OsGRF6/OsGRF10 module regulates the development of leaves and flowers [31]. In addition to the regulation of OsGRFs in growth and yield-related traits, the miR396b-OsGRF6 module can improve salt tolerance in rice by regulating the transcription factor MYB3R [32].

The extensively studied evolutionary relationships between Poaceae species could facilitate comparative studies of GRFs. The ancient whole-genome duplication events (e.g., τ-WGD, ρ-WGD) of the common ancestor of Poaceae grasses (including rice and sorghum) drastically shape the genomes of these grasses during their evolution [33,34]. Such evolutionary information of a certain gene family has been demonstrated to be valuable in reverse-genetic gene mining with the development of integrative gene duplication and genome-wide analysis (iGG) [35]. However, the evolutionary trajectory of *SbGRF*s remains in development, and their functional associations with biomass accumulation are almost unknown.

Here, we took sorghum as the research model to **(1)** re-identify the *SbGRF* family with emphasis on gene duplication and evolution; **(2)** identify *SbGRF* members functionally associated with internode elongation, maturation and metabolism of the cell wall and starch in the internode. The research focus was chosen because sorghum plants produce a large volume of biomass for forage/silage and biofuel production, and this feature has drawn extensive research attention for decades with biological relevance when compared with the related species, such as sugarcane, maize, and lemongrass [36,37,38,39,40,41,42,43,44]. Another reason is that sweet sorghum, as an understudied sorghum type, holds several important RNA-seq datasets to enhance our understanding of stem meristem maintenance, elongation, maturation and carbohydrate accumulation and allocation [37,38,39,45,46,47]. With the importance of stem tissue in sorghum biology, we combined genome-wide analysis, evolutionary comparison, comparative RNA-seq analysis and co-expression network approaches in this present study and discovered previously unknown functions of several SbGRFs in internode elongation, maturation and the potential involvement in the metabolism of cell wall and starch during post-anthesis stages.

## 2. Results and Discussion

### 2.1. SbGRF Family Is Expanded by Whole-Genome Duplication

**Figure 1:**
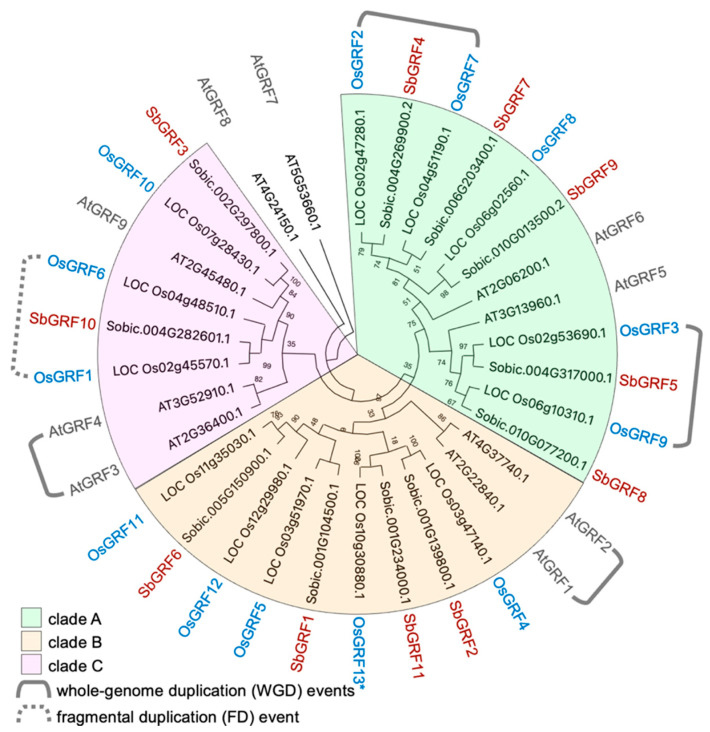
**Phylogenetic analysis of the SbGRF family**. OsGRFs, AtGRFs and SbGRFs were used in the phylogenetic analysis, and the GRF nomenclature was in accordance with the previous study [27]. Three phylogenetic clades of GRFs (i.e., clades A, B and C) were identified and are indicated in green, light yellow and pink colors, respectively. OsGRFs and AtGRFs that expanded due to the ancient whole-genome duplication events in monocots and dicots, respectively, are labeled with gray brackets. The OsGRFs that likely expanded from fragmental duplication are labeled with the gray dotted bracket.

Previous studies have reported the systematic identification of *GRF* genes from several Poaceae species, including *O. sativa*, *H. vulgare*, *B. distachyon*, *Z. mays* and *S. bicolor*, while evolutionary insights were discussed to emphasize those between the rice subspecies (e.g., ssp. *indica* and ssp. *japonica*) and its wild relative *O. rufipogon* [27]. Considering the large amount of biomass sorghum plants produced and the important roles of GRF as growth regulators, we sought to perform re-identification and detailed analyses of *SbGRF*s to obtain evolutionary insights into the *SbGRF* family and to address which *SbGRF*s may play important roles in sorghum stem growth. Genome-wide characterization identified 11 SbGRF-encoding genes. Phylogenetic analysis with the protein sequences together with both AtGRFs and OsGRFs separated these GRF proteins into three clades, clade A, clade B and clade C (presented in Figure 1 with green, light yellow and pink colors, respectively). Taking advantage of the established ancient whole-genome duplication (WGD) events that occurred in the Arabidopsis and rice genomes, respectively, and we identified both *AtGRF*s and *OsGRF*s were expanded mainly due to the WGD events [33,34]. Two pairs of *AtGRF*s derived from WGD events were found, while two pairs of *OsGRF*s were identified (labeled with gray brackets in Figure 1). Another pair of *OsGRF*s likely derived from fragmental or dispersed duplication was discovered (Figure 1 and Figure S1). In the phylogenetic tree constructed with the maximum-likelihood method, most of the SbGRFs were clustered well in one-to-one relationships with OsGRFs, demonstrating the conserved orthologous relationships between SbGRFs and OsGRFs. We further confirmed that our phylogenetically identified SbGRFs–OsGRFs relationships matched well with the previously reported orthologous connection between SbGRFs and OsGRFs determined with comparative genomic approaches [48]. Importantly, the WGD-derived *GRF* pairs were both retained in sorghum (*SbGRF4/7* and *SbGRF5/8*) and rice (*OsGRF2/7* and *OsGRF3/9*). Such retention of duplicated *GRF*s might have evolutionary importance, deserving further analyses.

### 2.2. Expression Analysis Suggests Functional Divergence of SbGRFs

**Figure 2:**
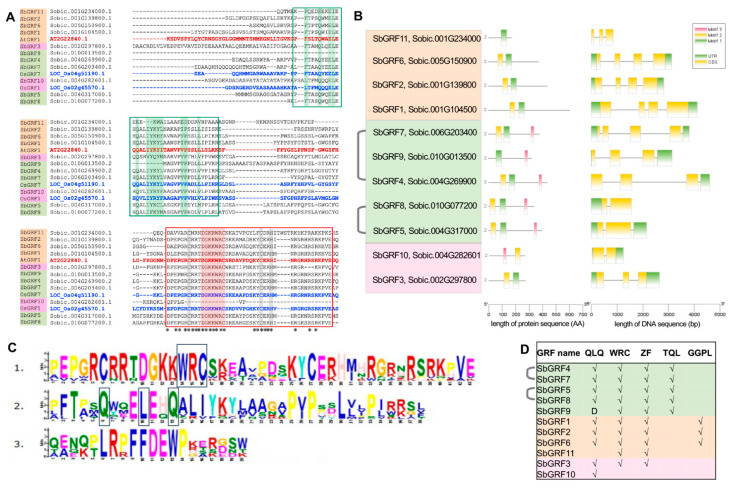
**Conservation of the GRF domains**. (**A**) The N-terminal regions of SbGRFs were aligned with the AtGRF1, OsGRF1 and OsGRF7, with AtGRF1 and OsGRF1/7 highlighted in red and blue fonts, respectively. The QLQ and WRC domains are indicated with green and red boxes with the highly conserved amino acid residues of these two domains highlighted in green and red backgrounds, respectively. The key amino acid residues for the CH zinc finger region are indicated in the gray background. (**B**) Analysis of the MEME-predicted protein motifs for SbGRF proteins and the gene structure analysis of *SbGRF* genes. (**C**) A detailed plot showing the MEME-predicted motifs 1, 2 and 3, while motifs 1 and 2 correspond to the WRC and QLQ domains, respectively. Black boxes indicate the conserved amino acid residues for the WRC and QLQ domains, respectively. (**D**) The table summarizing differences in domain and motif architectures between SbGRF proteins. “D” means a possible deletion causing a partial QLQ domain. The phylogenetic clade information of SbGRFs is indicated in background colors (green, light yellow and pink standing for clades A, B and C).

We explored the architecture of protein domains and motifs for SbGRFs and analyzed their expression patterns across multiple tissues and developmental stages in the reference cultivar BTx623. We sought to address whether the duplicated *SbGRF*s could become divergent in their encoded protein domains or gene expression patterns. GRF proteins typically contain two highly conserved domains: the QLQ and WRC domains, for which the QLQ domain is crucial for protein–protein interaction, while the WRC domain is important for DNA binding and putative nuclear localization [15]. A C3H zinc finger motif (abbreviated as ZF motif hereafter) also resides within the WRC domain, playing potential DNA binding ability to the GA-element [2,49]. We performed the alignment of SbGRF protein sequences together with representative AtGRF and OsGRF proteins and identified the QLQ and WRC domains and the ZF motif located at the N-terminal region of SbGRFs (Figure 2A). Further, we also identified the TQL and GGPL motifs that are found at the C-terminal of AtGRFs and potentially associated with transactivation ability [15,21] (Figure S2). MEME-based prediction of potentially conserved motifs found three motifs (designated as motifs 1, 2 and 3; Figure 2B), with motifs 1 and 2 matching the WRC and QLQ domains, respectively (Figure 2C). The MEME-predicted motif 3 contains a stretch of amino acid sequences (LRPFFDEWP) that are highly conserved among the SbGRFs, implying that this motif might be functional, deserving future investigations. Interestingly, we discovered on one hand that SbGRFs from different phylogenetic clades exhibit distinct domain architectures, with clade A SbGRFs containing the QLQ, WRC domains and the ZF and TQL motifs, while on the other hand, clade B SbGRFs contained the GGPL motif but lacked the TQL motif. This distinction in domain and motif architectures suggests the potential functional divergence of SbGRFs between the clades. On the other hand, SbGRFs encoded by the WGD *GRF* gene pairs (i.e., *SbGRF4/7* and *SbGRF5/8*) share the same domain and motif architecture, while the overall protein similarity was quite limited within each pair of the WGD-derived SbGRF proteins (ranging from ~50% to 60%) (Figure S3). The identical domain and motif architecture indicates that SbGRF4, 5, 7 and 8 might not become divergent in protein sequences.

**Figure 3:**
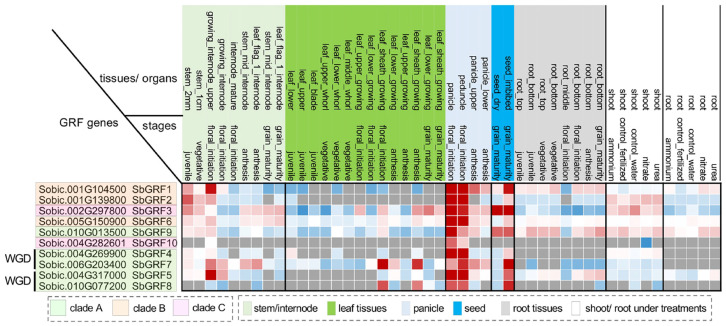
**Expression patterns of SbGRFs across multiple tissues and developmental stages in the sorghum cultivar BTx623**. Z-scores of the RNA-seq expression levels of SbGRFs are shown in the heat map (blue and red indicating low and high expression levels, respectively). Gray colors mean the gene was not expressed. The expression profiles are ordered first by the tissues/organs and then by stages.

Generally, the spatio–temporal expression preference of genes is well correlated to the function of genes [50]. Therefore, we investigated the expression profiles of *SbGRF*s across multiple tissues/organs and stages by using publicly available RNA-seq datasets [51]. *SbGRF*s exhibited different expression patterns between the phylogenetic clades. For example, clade A *SbGRF*s showed stronger tissue/organ expression preferences than those of the other clades, mainly expressed in the shoot, stem, panicle and seeds (Figure 3). *SbGRF1*, *3*, *6* and *9* have relatively wide expression patterns at multiple tissues and organs (e.g., stems, leaves, panicles, seeds, roots and shoots). Particularly, *SbGRF10* has the highest spatio–temporal expression specificity, only detected in the growing upper internodes and growing panicles and peduncles during flower initiation. Such a specific expression pattern in the growing stem and panicle suggests *SbGRF10* may have a specific role in the growing tissue, deserving future functional characterization.

With this BTx623 expression atlas, we reckoned whether the WGD SbGRF pairs could share similar or different expression profiles. We found that SbGRF4 and 7 were expressed in distinct ways. SbGRF4 has higher expression levels at the panicles followed by the shoot tissue and growing stem. By contrast, SbGRF7 was expressed mostly at the later stages of the stems and panicles but had low expression levels at the shoot. For the other WGD pair SbGRF5/8, SbGRF8 was specifically expressed in the leaf sheath, panicle and seeds, while SbGRF5 was widely expressed at the stem, leaf, panicle, seeds, root and shoot, with higher expression levels at the growing internodes and roots than that of SbGRF8. In short, our expression analysis demonstrates that the WGD-derived SbGRF pairs could become functionally divergent due to differential expression patterns, rather than adopting distinct domain and motif architectures.

To further address whether the WGD-duplicated *OsGRF* pairs could have similar expression patterns when compared with the *SbGRF* orthologs, we studied the tissue expression preference of *OsGRF*s by using the IC4R rice expression atlas [52]. First, we compared if the duplicated *OsGRF* pairs (*OsGRF2/7*, *OsGRF3/9* and *OsGRF1/6*) could have similar expression patterns (Figure S4). Our analysis demonstrated that *OsGRF2* and *7* share a very similar tissue expression preference between each other, while the same observation was seen for *OsGRF3* and *9*. Similarly, the fragmental duplicated *OsGRF1* and *6* have similar expression patterns, except that *OsGRF6*, but not *OsGRF1*, was expressed in the callus (Figure S4). Overall, the expression analysis supports that duplicated *OsGRF* pairs may possibly have redundant functions, except for in certain tissues or under certain conditions. Notably, the IC4R collects and analyzes rice RNA-seq expression data sets from different labs, different batches, treatments and experimental conditions, thus the rice expression data provided in the IC4R should not be suitable for a direct comparison with the expression atlas of sorghum cultivar BTx623. However, as the rice IC4R expression atlas compiles and unifies multiple rice RNA-seq datasets and plots the expression levels according to tissues (i.e., aleurone, anther, callus, leaf, panicle, seed root, pistil and shoot), this data layout allows for a direct comparison of the tissue expression preference between *OsGRF*s and the orthologous *SbGRF*s, especially in the leaf, panicle, seed and root tissues (Figure S4B). We therefore evaluated whether the tissue expression preference of *OsGRF* between leaf, panicle, seed and root tissues could be similar to that of the corresponding *SbGRF* ortholog. We found that *SbGRF4*’s tissue expression preference may be similar to that of *OsGRF2*, while *SbGRF8*’s expression pattern may be similar to that of *OsGRF9* as well. This similarity of tissue expression preference was also seen between *SbGRF3* and *OsGRF10* and *SbGRF10* and *OsGRF1*. These results imply that the above-mentioned *SbGRF* and corresponding *OsGRF* pairs might have similar, at least partially, biological functions. However, we already demonstrated that the WGD pairs *SbGRF4/7* and *SbGRF5/8* displayed different expression profiles within each pair (Figure 3), which is in contrast to the observation for the rice WGD pairs, *OsGRF2/7* and *OsGRF3/9* (Figure S4). This finding serves as evidence to support that, after the ancient whole-genome duplication events of these *GRF*s, the duplicated *OsGRF*s and *SbGRF*s, respectively, could experience distinct evolutionary trajectories and selection, which have led to the obvious expression divergence between *SbGRF4* and *7* or *SbGRF5* and *8*.

### 2.3. Analyses of SbGRF Expression Patterns in Sorghum Internodes Identifies Their Roles in Internode Elongation and Metabolism

**Figure 4:**
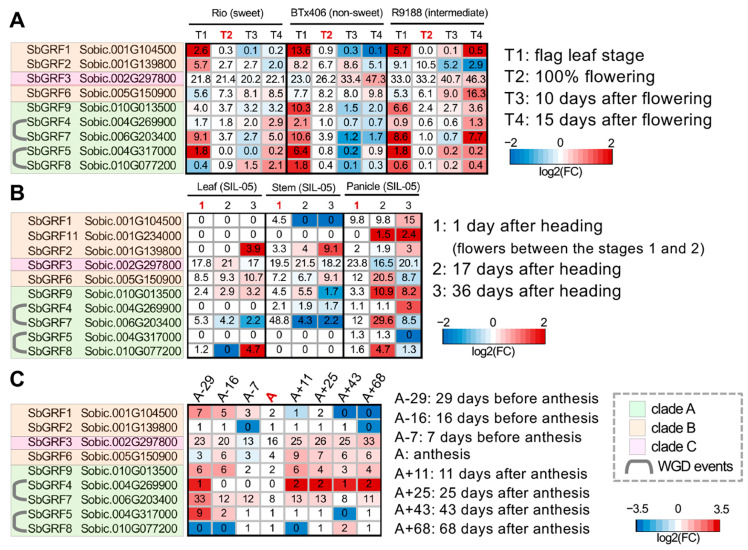
**Comparative expression analysis of *SbGRF*s in the internode among multiple sorghum cultivars.** In each figure, the colors of the heat map reflect the magnitude of the log2 fold change of gene expression levels relative to the corresponding anthesis stage (indicated in bold and red fonts) per tissue type per cultivar. For Figure 4B, because the RNA-seq dataset only sampled the heading stage rather than the anthesis stage, the gene expression values at the second and third stages of the leaf, stem and panicle tissues, respectively, were compared with the heading stage of the corresponding tissue. The absolute expression levels (in RPKM) are labeled on each cell of the heatmaps. The internode stages are labeled on each figure, with the anthesis stage highlighted with red fonts in figures A and C. Particularly, the heading stage is highlighted with red fonts for figure B. (**A**) The expression profiles of *SbGRF*s at the T1, T2, T3 and T4 stages in the internodes from sweet sorghum Rio, grain sorghum BTx406 and the introgression line R9188; the phenotype of internode sugar accumulation is briefly indicated in brackets on the figure. (**B**) The expression profiles of *SbGRF*s at the 1 DAH, 17 DAH and 36 DAH stages collected from leaf, stem and panicle tissues from the Japanese sweet sorghum SIL-05; (**C**) The expression profiles of *SbGRF*s at a series of time courses before and after anthesis in the internode of sweet sorghum Della.

In contrast to the widely reported roles of AtGRFs in different aspects of leaf growth, we noticed that a number of *SbGRF*s had high expression levels in the stem or internode, indicating previously unknown roles of these growth regulators during the process of stem elongation and/or maturation. Sorghum stems represent a key component of its biomass yield and are agro–economically important as sweet sorghum stems accumulate soluble sugar and a relatively high amount of starch [46]. Previous RNA-seq datasets have established the resources for mining the genes that could be involved in stem elongation, development, maturation and post-elongation metabolism in several sweet sorghum cultivars (i.e., Rio, Della and SIL-05) [37,38,39,45]. Our expression analysis identified that *SbGRF*s possess several different expression patterns. First, *SbGRF1* was expressed before heading and anthesis, but its expression level was decreased to very low levels possibly after heading in both Rio, Della and SIL-05 (Figure 4). Second, a group of *SbGRF*s (*SbGRF5* and *7*) were down-regulated along with the internode elongation process and had the lowest expression levels at anthesis. After flowering, *SbGRF7* was up-regulated or maintained its expression in the internode, suggesting a potential role in internode maturation and/or metabolism. Sorghum internodes finish the elongation process before heading, which represents a stage between flag leaf emergence and flowering (labeled T1 and T2, respectively, in Figure 4A). By then, the cell elongation and expansion should be complete in the internode [37]. Thus, the *SbGRF*s that remain stably expressed in the internode could possibly have previously unknown roles in maintaining cell wall homeostasis and/or the metabolism of carbohydrates. Indeed, we noticed that *SbGRF3/4/6/9* have relatively stable expression patterns across the time series in the internodes of Rio, Della and SIL-05 (Figure 4). While these *SbGRF*s have a lower expression at anthesis, their expression levels were raised back during post-anthesis stages. It is worth noting that some *SbGRF*s have different expression patterns between the cultivars, possibly reflecting technical differences between the RNA-seq experiments (e.g., the data output of RNA-seq, parameters in the RNA-seq analysis pipeline) or cultivar-specific expression variations.

In addition, our RNA-seq data includes three genetically related accessions, Rio, BTx406 and their introgression line R9188 (described in the Methods section). These three cultivars have contrasting sugar-accumulation phenotypes in their internodes. Rio is a typical sweet sorghum cultivar with high sugar content in the internode after flowering, while the introgression line R9188 has high sugar contents around flowering but could not maintain the high-sugar phenotype, with BTx406 being a low-stem sugar cultivar [38]. The RNA-seq data of Rio/BTx406/R9188 further validated that several *SbGRF*s (*SbGRF1*, *5* and *7*) were decreased along with the internode elongation (from the T1 to T2 stage), suggesting their functional associations with cell proliferation and/or elongation and stem growth (Figure 4A). In addition, we observed that some *SbGRF*s (*SbGRF4*, *6*, *9*) had a gradual decrease between the stages or a relatively stable expression level, indicating their functions might be related to post-anthesis internode maturation and carbohydrate metabolism. Interestingly, *SbGRF3*, being the one with the highest expression level in the *SbGRF* family, was stably expressed in Rio but up-regulated in BTx406 and R9188, implying that SbGRF3 expression could be related to the repression of carbohydrate metabolism (including sucrose and starch) (Figure 4A) [38]. Briefly, a comparison of gene expression across genetically related cultivars confirmed the distinct expression patterns between SbGRF genes with SbGRF3 being associated with the regulation of carbohydrate metabolism.

**Figure 5:**
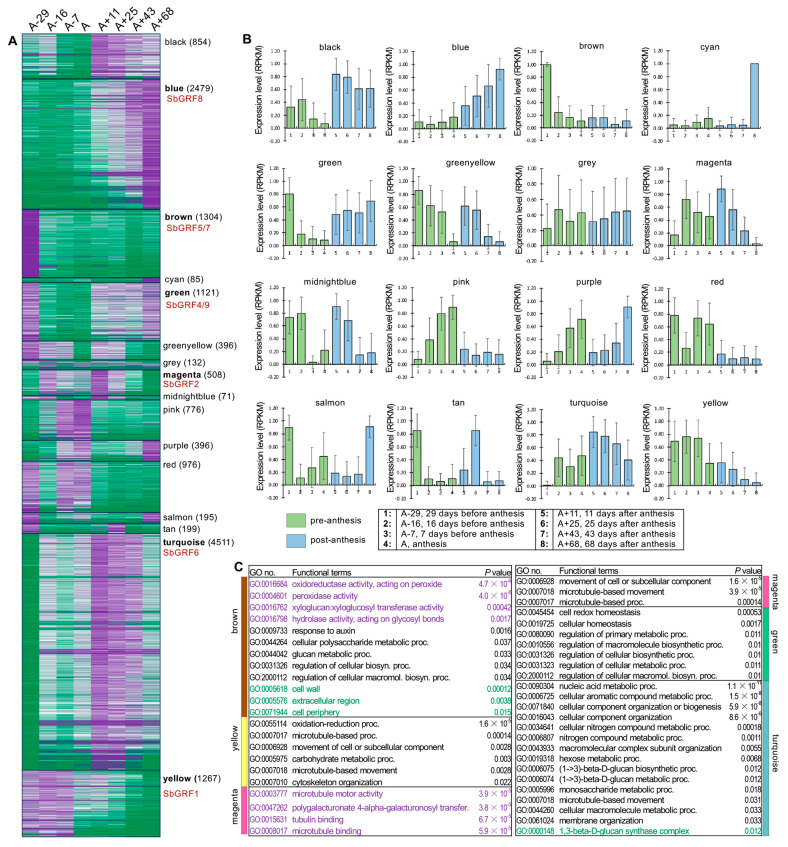
Co-expression analysis of the time-series sorghum RNA-seq data identified the SbGRFs associated with internode elongation and cell wall modification at the post-anthesis stages. (**A**) The heat map showing the 15 co-expression modules identified with WGCNA. The gray module represents the group of genes that do not share significant co-expression patterns. The module names are labeled, with the total number of genes per module labeled in brackets and the selected SbGRFs within the modules indicated in red. (**B**) Representative expression patterns (eigengenes) of the 15 co-expressed modules and the gray-module genes. The pre-anthesis and anthesis stages are presented with green bars, while the post-anthesis stages are presented with blue bars. The error bars indicate the standard deviation of eigengene expression. The eight stages of the Della internode were abbreviated as A−29, A−16, A−7, A, A+11, A+25, A+43 and A+68, respectively. A−29, 29 days before anthesis; A−16, 16 days before anthesis; A−7, 7 days before anthesis. A, anthesis; A+11, 11 days after anthesis; A+25, 25 days after anthesis; A+43, 43 days after anthesis; A+68, 68 days after anthesis. (**C**) The GO functional terms enriched in the SbGRF-containing co-expression modules (i.e., the brown, green, magenta, turquoise and yellow modules), with the GO term numbers and term descriptions and P values provided. The molecular function GO terms, biological process terms and cellular component terms are indicated in purple, black and green fonts, respectively.

To pinpoint the roles of SbGRFs during the process of internode elongation and post-maturation, we constructed the co-expression networks for a total of 17,116 differentially expressed genes in the Della internode transcriptome data [37,39]. The weighted gene co-expression network analysis (WGCNA) approach identified 15 co-expression modules with the gray module consisting of several hundreds of genes that cannot be grouped into any co-expression modules (Figure S5, Table S1). Visualization of the expression patterns of these co-expression modules highlighted that anthesis is associated with large-scale transcriptome reprogramming in the internode with dramatic changes in gene expression detected between the pre-anthesis and post-anthesis stages (i.e., the A−29 to A stages versus the A+11 to A+68 stages) (Figure 5A,B). *SbGRFs* fell into six co-expression modules (i.e., the blue, brown, green, magenta, turquoise and yellow modules). Since *SbGRF2* has the lowest expression level, we focused on the other co-expression modules containing differentially expressed *SbGRF*s. Interestingly, two *SbGRF*-containing modules (the brown and yellow modules) exhibited declined trends of expression, with the expression patterns of the brown module genes being dramatically decreased after internode elongation (the A−29 stage). Differing from the brown module, the genes from the yellow module were sharply down-regulated after the A−7 stage, suggesting a downregulation of expression associated with flowering (Figure 5B). In contrast to the brown and yellow modules showing declined trends, the blue and green modules (containing 2479 and 1121 genes, respectively) exhibited gradually increasing trends of gene expression. In addition, genes from the black module were downregulated before anthesis and up-regulated right after anthesis, with the highest expression levels detected at the A+11 stage. Since the blue and green modules contain *SbGRF*s and were up-regulated during the post-anthesis stages, we focused on these two modules hereafter. The green module contains *SbGRF4* and *9* and was decreased from the A−16 to anthesis stages but dramatically up-regulated during post-anthesis stages. Further, the turquoise module containing *SbGRF6* also displayed relatively higher expression levels at the post-anthesis stages than those at the pre-anthesis stages. We conducted gene ontology (GO) enrichment analysis for the *SbGRF*-containing modules in order to enhance the understanding of *SbGRF*-associated biological functions (Figure 5C, Table S2). Interestingly, no matter whether the *SbGRF*-containing modules show up-regulation or down-regulation expression trends during the post-anthesis stages, these modules are all enriched by cell wall metabolism-related functional terms, such as the cellular polysaccharide metabolic process, glucan metabolic process and regulation of cellular biosynthesis process. Collectively, this co-expression network analysis of transcriptome data of the sweet sorghum internodes suggests *SbGRF*s exhibit several types of expression profiles during the reproductive stages in internodes that are associated with cell wall metabolic functions.

**Figure 6:**
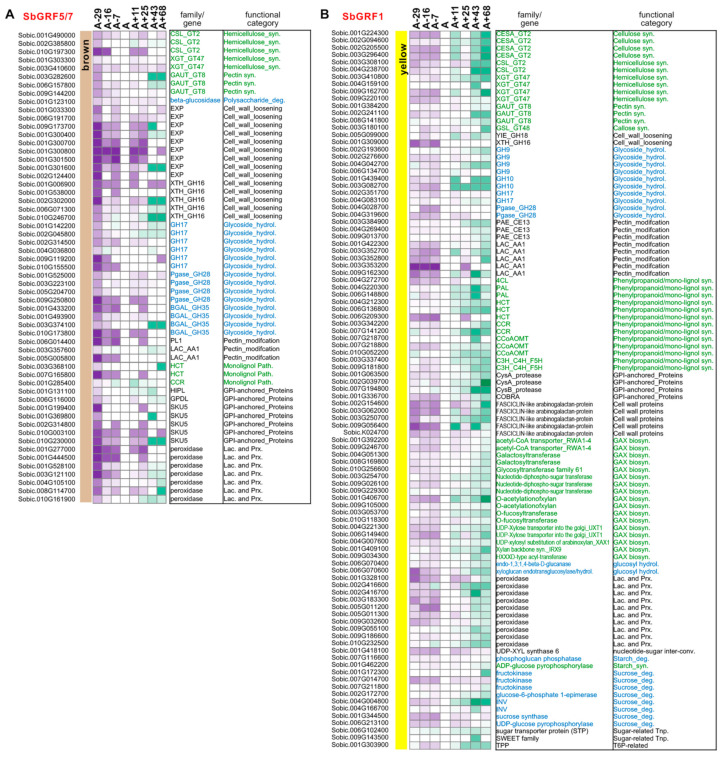
The expression patterns of select carbohydrate metabolic genes in the brown (**A**) and yellow (**B**) modules of the Della co-expression network. The representative carbohydrate metabolic genes were selected to show their expression profiles based on the gene annotations reported [37,38,53]. Expression patterns of the carbohydrate metabolic genes in the SbGRF-containing modules with declining trends (i.e., the brown and yellow modules) are shown in the figure. Gene expression dynamics are shown in heatmaps and the colors are shaded to reflect the magnitude of log2 fold change of gene expression relative to the anthesis stage in each co-expression network. The sorghum geneIDs are given on the left side of the heat map, while the gene annotation and their functional categories are provided on the right side of the heat map, with the biosynthetic and degradation genes shown in green and blue fonts, respectively.

To gain further insights into the potential roles of SbGRFs in the internode during post-reproductive stages, we took advantage of the well-annotated cell wall metabolic genes reported in several previous studies, which cover a wide range of cell wall metabolic aspects, such as the biosynthesis of primary and secondary cell wall components (e.g., cellulose, hemicellulose, pectin, mono-lignol, lignin, and callose) and the cell wall loosening, reassembly and controlled degradation [37,38,39,53]. In the brown module with a sharply decreased expression after the internode-elongating A−29 stage, numerous genes were dramatically down-regulated after the A−29 stage (Figure 6A), which function in hemicellulose biosynthesis (e.g., cellulose synthase-like (CSL), xyloglucan galactosyltransferases (XGTs)), pectin biosynthesis (homogalacturonan α-1,4-galacturonosyltransferases, GAUT), and mono-lignol synthesis (cinnamoyl CoA reductase, CCR; hydroxycinnamoyl-CoA transferase, HCT), as well as cell wall loosening and expansion (e.g., expansins (EXP), xyloglucan endotransglucosylases/hydrolases (XTH) and GPI-anchored proteins). The two CSLs (Sobic.001G490000 and Sobic.002G385800) in the brown module are homologous to OsCSLA4 and OsCSLA7, which are required for mannan or glucomannan synthesis [46,47,48,54,55,56]. A number of XTH genes were decreased after internode elongation, and potentially involved in cell wall remodeling and loosening [57,58]. In the yellow module, a large number of genes involved in secondary cell wall (SCW) synthesis were down-regulated, with a gradually decreasing trend during post-anthesis (Figure 6B). These genes include several cellulose synthase A-encoding genes for SCW (Sobic.003G296400 (CESA4), Sobic.001G224300 (CESA7), Sobic.002G205500 (CESA9)), CSL genes for hemicellulose synthesis, GAUT genes for pectin synthesis and PAL, 4CL, HCT, CCR, CCoAOMT, C3H, C4H and F5H genes for phenylpropanoid and mono-lignol biosynthetic pathways. Also, five genes encoding FASCICLIN-like arabinogalactan proteins were down-regulated in this module, which has been reported to hold important roles in SCW formation [59,60,61]. Previous analyses have highlighted the complexity of multiple gene family expression profiles during internode elongation and thereafter, reflecting the presence of numerous cell types in sorghum stems (such as epidermal, fiber cells, xylem, phloem and pith parenchyma cells) [37,62]. In our analyses, the brown and yellow modules with distinct patterns of gene down-regulation possibly represent two major groups of cell types that may stop proliferation and/or expansion after internode elongation.

**Figure 7:**
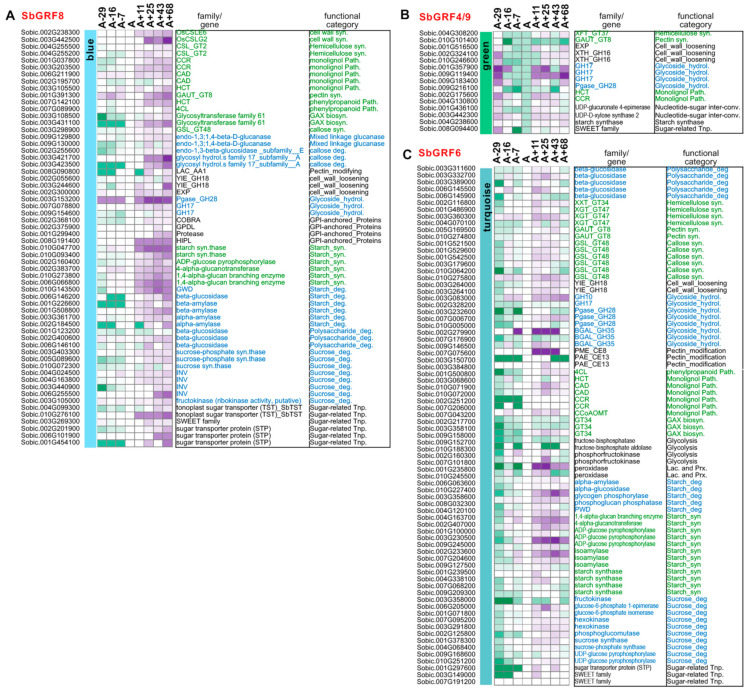
The expression patterns of select carbohydrate metabolic genes in the blue (**A**), green (**B**) and turquoise (**C**) modules of the Della co-expression network. The representative carbohydrate metabolic genes were selected to show their expression profiles based on the gene annotations reported [37,38,53]. Expression patterns of the carbohydrate metabolic genes in the SbGRF-containing modules with increasing trends (i.e., the blue, green and turquoise modules) are shown in the figure. Gene expression dynamics are shown in heatmaps and the colors are shaded to reflect the magnitude of the log2 fold change of gene expression relative to the anthesis stage in each co-expression network. The sorghum geneIDs are given on the left side of the heat map, while the gene annotation and their functional categories are provided on the right side of the heat map, with the biosynthetic and degradation genes shown in green and blue fonts, respectively.

Many genes related to the metabolism of cell wall components and starch were found in the modules with up-regulation expression trends (i.e., blue, green and turquoise). In the green module, a few genes functioning in hemicellulose synthesis (xyloglucan fucosyltransferase, XFT; GAUT), cell wall loosening (EXP and XTH), and monolignol synthesis (HCT and CCR) were up-regulated after the anthesis stage (Figure 7). The expression levels of two genes working on the nucleotide–sugar interconversion were raised after flowering. The nucleotide–sugar interconversion pathway is important for generating substrates for cell wall biosynthesis [63]. The two up-regulated modules (the blue and turquoise modules) during the post-anthesis stages do not only contain cell wall metabolic genes but also contain starch metabolic genes, consisting of the characteristics of sweet sorghum, in which the internode usually has higher amounts of starch than those of the grain sorghum cultivars [46]. The turquoise module contains SbGRF6 and numerous genes related to SCW formation, such as the biosynthesis of hemicellulose, pectin, callose and mono-lignols (e.g., XXT, XGT, GAUT, GSL, 4CL, HCT, CAD, CCR and CCoAOMT). Similarly, pectin modification and cell wall loosening-related genes, such as pectin acetyl esterases (PAE), pectin methyl esterases (PME) and yieldins (YIE), were up-regulated in the module. These pectin-modifying enzymes are important for cell wall loosening and reassembly [53]. Grass cell walls contain significant amounts of glucuronoarabinoxylan (GAX) [63,64], and GX biosynthesis is involved in several gene families, including the GT8 (GAUT), GT31, GT34 (XXT), GT47 (XGT) and GT61 families [63,65]. Thus, the GAX biosynthesis may be regulated during the post-anthesis stages as well.

In the blue module, the up-regulated cell wall metabolic genes include those involved in cellulose synthesis (the homologs of OsCESA2 and OsCSLE6) and the synthesis of hemicellulose, GAX and monolignol. Interestingly, genes encoding the SCW-cellulose synthase and several proposed metabolic flux regulators of the phenylpropanoid biosynthesis (i.e., L-Phe/-Tyr ammonia-lyases, SbPTALs (Sobic.004G220300, Sobic.006G148800) and phenylalanine ammonia-lyase, SbPAL (Sobic.004G220400)) were not significantly up-regulated and grouped into the blue and/or turquoise modules [66]. By contrast, many genes encoding enzymes for degrading polysaccharides or cell wall components were detected in these modules, including mixed linkage glucanase, glycoside hydrolases (mainly belonging to the GH17, GH28 and GH35 families) and beta-glucosidase. Considering the up-regulation of nucleotide–sugar interconversion enzymes in the green module, these expression patterns suggest that there might not be enough carbon flux from the biosynthetic pathways directed by the SbPTALs/SbPALs at these post-anthesis stages, and the main sources for cell wall synthesis and maintenance might come from re-utilization of other degrading carbohydrates (for instance, the conversion of soluble sugars, degradation from starch or other cell wall components from other cell types). Meanwhile, we observed numerous genes that are mainly involved in starch synthesis and degradation are up-regulated during the post-anthesis stages, indicating that starch contents may be increased and starch homeostasis could be delicately controlled. In the blue and turquoise modules, the genes encoding both glycosyl hydrolases family 17 proteins and callose synthase (also known as 1,3-beta-glucan synthase) were identified to be responsible for callose degradation and synthesis, respectively, indicating that callose turnover possibly represents important regulation of the homeostasis of polysaccharide carbohydrates [37]. It has been known that callose turnover could play a role in phloem development [67]. Further, the cell walls of some grass species (like rice and sorghum) contain a considerable proportion of (1,3;1,4)-beta-D-glucans (MLG), which has been reported as a cell wall component in certain tissues or organs, such as seeds and mature stems [68,69]. Consistent with our finding that during the post-anthesis stages the major sources of carbon for cell wall maintenance (including the substrates for cell wall synthesis like UDPG and mono-lignols) may come from the degradation or homeostasis from some cell wall components or polysaccharides, rather than from de novo biosynthesis, we observed that the degradative enzymes for MLG were up-regulated. Previous studies have considered that the enzymes for MLG degradation (e.g., mixed linkage glucanases, b-galactosidases) could be involved in MLG turnover and be important for cell wall remodeling [70,71,72]. Similarly, several polysaccharide-degrading enzymes (e.g., Pgase and BGALs) and pectin modification (e.g., PAEs, PMEs) that are key to cell wall reassembly and degradation were coordinately up-regulated from the A+11 to A+68 stages. These lines of gene expression evidence support that cell wall reassembly and degradation might be crucial for the metabolism and maturation of internodes during post-anthesis stages. More recently, a detailed RNA-seq study analyzing the intercalary meristem of young sorghum stems reveals the gene regulatory networks and highlights the roles of hormone signaling (including auxin, gibberellic acid (GA), cytokinin and brassinosteroid) and several transcription factors (including GRFs and ARFs) in cell proliferation and differentiation during stem growth [47]. As a major GA-responsive growth regulator, whether GRF family members play a role after the cessation of stem elongation remains an important biological question. By integrative multiple RNA-seq datasets and co-expression network analysis, we provide evidence to support that SbGRFs could be separated into different modules according to expression patterns, and they might be involved in the regulation of biosynthesis of cell wall components and cell wall loosening and reassembly. Cell wall reassembly may be essential for sweet sorghum internode maturation because soluble sugars are accumulated in the internode during post-anthesis stages, while the internode water contents are gradually decreased with starch accumulation [38,39,46]. To accommodate the physiological and metabolic changes (e.g., the water potential of the cells, carbon availability) during the post-anthesis stages, transcriptional coordination of carbon sink strength was identified previously, yet the potential regulators have not been studied [38]. In this present study, the RNA-seq analyses found that SbGRF1, 5 and 7 could be involved in the down-regulation of the biosynthesis of cell wall components, while SbGRF4, 6, 8 and 9 could regulate cell wall loosening, reassembly and/or the associated starch synthesis.

Co-expression network analysis is a key approach for us to understand the potential functions of *SbGRF*s in our work. Co-expression networks help link known homology-based genes with unknowns through a “guilt-by-association” approach [73]. Considering the fact that a relatively small proportion of the genes have been functionally characterized even in the best-studied plant species (for instance, ~4100 out of the ~35,000 annotated genes have been characterized in rice), a combination of evolutionary analysis and co-expression networks could further unleash the power to understand the conservation and divergence of genes and gene families [74]. In our research work, co-expression analysis can identify functional gene modules and enhance our knowledge of the *GRF* family. A recent advance further demonstrates the power of using co-expression networks in an evolutionary context. A comprehensive co-expression network of the closest algal relatives of land plants (*Mesotaenium endlicherianum*) has been compared with those obtained from alga and land plants, such as *Zygnema circumcarinatum*, *Physcomitrium patens* and *Arabidopsis thaliana*, revealing ancient genetic programs and conserved hubs across several million years of evolution [75]. Similarly, a wheat and rice co-expression network analysis focused on the famous drought regulator dehydration-responsive element-binding (DREB) proteins identifies a DREB member ERF014 that co-evolves with a small heat shock protein cluster to regulate heat response in wheat [76]. These works exemplify that the combination of co-expression networks and evolutionary analyses could identify not only the conserved genetic programs and regulatory hubs but also recognize those molecular regulators of which sub-functionalization or neo-functionalization has occurred. Given limitations in gene functional characterization in under-studied species (e.g., difficulty in transformation and limited availability of mutant lines), a combination of co-expression network and evolutionary analyses should be of great promise to explore gene functions.

## 3. Materials and Methods

### 3.1. Genome-Wide Identification and Sequence Analysis of Sorghum GRFs

To comprehensively identify the GRF proteins and their encoding genes, a combinatory approach using both the protein-domain searching method and the BLAST-blast method was used. Rice (*Oryza sativa*) GRF proteins in the Nipponbare genome v7 and the Arabidopsis GRF proteins are in accordance with previous studies and are used for the BLAST identification of sorghum GRFs (E-value < 1 × 10^−10^) [2,15,21]. The *Sorghum bicolor* reference genome of the cv. BTx623 v3.1.1 was used [51]. For protein-domain searching, the hidden Markov model (HMM) profiles of the QLQ domain (PF08880) and the WRC domain (PF08879) were used for the Simple HMM Search program in the TBtools-II v2.110 software against all of the protein sequences of the sorghum BTx623 genome [77]. The identified proteins were further validated using the Conserved Domain Database (v3.21) of the NCBI and the SMART database (v5.69) (CDD and SMART databases accessed 20 July 2024) [78,79]. Finally, the proteins and the corresponding encoding genes identified from both of the methods were confirmed as SbGRFs. To simplify the phylogenetic and downstream analysis, only the predicted proteins encoded by the primary transcript of the *SbGRF* genes were used.

Multiple sequence alignments of the GRF proteins were performed with the MUSCLE program (v5) [80]. Conservative motifs were predicted with the MEME tool v5.1.1 [81]. The percentages of sequence identities between the SbGRF and OsGRF proteins were calculated using the “Percent Identity Matrix” function of the Clustal-Omega tool from the EMBL-EBI website (https://www.ebi.ac.uk/Tools/msa/clustalo/ (accessed on 1 June 2024)) [82]. The SbGRF–OsGRF protein identity matrix could facilitate the identification of duplicated SbGRF copies [35].

### 3.2. Phylogenetic Analysis Sorghum GRFs

The alignment of the protein sequences of SbGRFs, AtGRFs and OsGRFs was used to construct a phylogenetic tree with the maximum-likelihood method (ML) with 1000 bootstrap replicates by using the MEGA-X software [83]. To interpret the phylogeny of GRF proteins, the ancient whole-genome duplication events that occurred in *Arabidopsis thaliana* (namely, α, β, γ-WGD) and in the common ancestor of Poaceae species (namely, ρ-WGD), respectively, were used in this present study [33,34,84]. In particular, the rice gene model and its derived protein (LOC_Os10g30880, namely OsGRF13* herein; * highlights that it is not considered a regular GRF protein) was included in the phylogenetic analysis to better reflect the evolutionary relationships between the genes potentially encoding GRF proteins, even though OsGRF13* cannot be categorized in the GRF family because the N-terminal conserved QLQ domain of OsGRF13* is deleted [15].

### 3.3. Gene Expression Analysis of SbGRFs

To capture the expression patterns across multiple tissues, organs and developmental stages, several RNA-seq data sets were used. First, the sorghum BTx623 expression atlas, including the leaf tissues (leaf blade and leaf sheath), stems (including growing stems and certain internodes), panicles, seeds, roots and shoots, was used to understand the tissues/organs and stages when and where each *SbGRF* is expressed [51]. Since the BTx623 expression atlas includes multiple stages for each type of tissue or organ, the rice expression atlas from the IC4R database was used as a comparable gene expression resource to evaluate whether the *OsGRF* orthologous genes could have similar tissue/organ expression preferences with the corresponding *SbGRF*s [52].

Since sweet sorghum cultivars represent an important type of varieties of sorghum breeding and agricultural applications, a set of comparative RNA-seq data using three genetically related but phenotypically contrasting sorghum lines (Rio, BTx406, R9188) was used in our analysis [38]. Among the three cultivars, Rio is a typical sweet sorghum cultivar widely used in the U.S.A., with its stems accumulating high levels of soluble sugar and relatively higher amounts of starch compared to grain sorghum cultivars [46,85], while BTx406 is a classical grain sorghum with a dwarf plant stature that accumulates low levels of sugar in its stems. R9188 is a sweet sorghum-converted line with a mostly Rio genetic background. The comparable upper internodes of Rio, BTx406 and R9188, respectively, were collected in the field trial at four stages (designated T1, T2, T3 and T4, corresponding to the flag leaf stage, flowering stage, 10 days after flowering and 15 days after flowering, respectively) and their RNA-seq analyses have been reported with the co-expression network established [38].

In addition, Della, another representative sweet sorghum cultivar, and SIL-05, a Japanese sweet sorghum line with internode transcriptome data sets available, were used in our work. The time-series RNA-seq data of Della internodes ranged from floral induction and anthesis to complete grain maturity, with the stages designated A−29, A−16, A−7, A, A+11, A+25, A+43 and A+68 (“A” standing for anthesis) [37]. During the plant development of Della, floral induction happens at the A−29 stage, while grains become mature around 30 days post-anthesis, with the A+43 and A+68 representing post-grain-maturity stages. When grains become mature (after the A+25 stage), the starch content in the internode is dramatically increased [46]. For the Japanese sweet sorghum SIL-05, the stem, leaf and panicle tissues were sampled at one day, 17 days and 36 days after heading, respectively (abbreviated as 1 DAH, 17DAH and 36 DAH), for RNA-seq analysis [45]. This SIL-05 transcriptome data thus do not only provide another independent sorghum internode expression data set but also allow for the comparison of gene expression between the three tissues. Previous comparative studies have demonstrated that these three RNA-seq datasets focused on sorghum internodes are comparable and deliver novel insights into the common expression networks coordinating carbohydrate metabolism [38]. In this present study, to avoid potential batch effects and other technical artifacts, gene expression profiles were not compared across the three RNA-seq datasets but limited the comparison within a dataset, with emphases on the magnitude of fold changes relative to the anthesis stage. Particularly, since the SIL-05 RNA-seq did not capture the anthesis stage, the expression profiles were compared to the 1DAH stage. RNA-seq statistics, quality control and differentially expressed genes for each of the three RNA-seq datasets have been reported elsewhere [38,39,45].

Co-expression networks have been constructed with the weighted gene co-expression network analysis (WGCNA v1.72-5) method for the Della internode transcriptome data. Briefly, the correlation values between gene expression profiles were calculated using the robust bi-weight–mid-correlation method and raised to a soft threshold power, in which the co-expression network was fitted to a scale-free topology [86,87] (Figure S5). Then, a signed hybrid weighted correlation network was constructed to identify modules of interconnected genes (with a minimum module size of 50 genes) with high topological overlap (TO). A co-expression module captures a group of genes with interconnected expression patterns, and those closely related modules with eigengene expression correlation > 0.9 were merged as one module. To obtain representative biological processes for each co-expressed module, functional enrichment analysis was performed with the Gene Ontology (GO) annotation by using the ClusterProfiler v4.0 package (*P_hypergeometric_* < 0.05) [88].

## 4. Conclusions

In summary, we resolve the 11 *SbGRF*s into three phylogenetic groups and interpret their evolutionary trajectories. We provide evidence to support the divergence of WGD-duplicated *SbGRF* pairs. With the co-expression analysis, *SbGRF 1*, *5* and *7* could be involved in the down-regulation of the biosynthesis of cell wall components, while *SbGRF 4*, *6*, *8* and *9* could be associated with the regulation of cell wall loosening, reassembly and/or starch biosynthesis. Given the potential new roles of SbGRFs in internode elongation, maturation and metabolism, our findings presented here warrant experimental investigations in the future to elucidate the genetic effects of these internode-related SbGRFs and their regulation with downstream genes with modern molecular genetic methods.

## Data Availability

The data presented in the study are available in the article and the Supplementary Materials. For further inquiries, you can contact the corresponding author directly.

## Supplementary Materials

The following supporting information can be downloaded at: https://www.mdpi.com/article/10.3390/plants13172352/s1. The following supporting materials are available with the paper itself: Figure S1: Identification of duplicated SbGRF gene pairs with the MCscanX software; Figure S2: Alignment of the full-length protein sequences of SbGRFs; Figure S3: The similarity matrix between SbGRF proteins; Figure S4: The gene expression patterns of OsGRFs and the evaluation on the expression similarities between OsGRFs; Figure S5: Construction of the co-expression networks with the Della internode RNA-seq data; Table S1: WGCNA identified co-expression networks of gene expression in the Della internodes. Table S2: The GO functional terms enriched in the SbGRF-containing modules of the Della co-expression networks.
