# Supplementary material for: Potential Roles of the GRF Transcription Factors in Sorghum Internodes during Post-Reproductive Stages
Source: Plants (Basel). 2024 Aug 23;13(17):2352. doi: 10.3390/plants13172352 (PMC11396856; doi:10.3390/plants13172352)
Supplement: Supplementary file 1 [file plants-13-02352-s001.zip › Mint_SbGRF_supplV3_20240801.pdf]

# Supplementary information
# Potential roles of the GRF transcription factors in sorghum internodes during post-reproductive stages

Min Tu [1,*,†], Zhuang Li [1,†], Yuanlin Zhu [1], Peng Wang [2], Hongbin Jia [1], Guoli Wang [3], Qin Zhou [1], Yuqing Hua [1], Lin Yang [1], Jiangrong Xiao [1], Guangsen Song [1] and Yin Li [3,*]

[1] Hubei Technical Engineering Research Center for Chemical Utilization and Engineering Development of Agricultural and Byproduct Resources, School of Chemical and Environmental Engineering, Wuhan Polytechnic University, Wuhan 430023, China; 15826790260@163.com (Z.L.); jlsyzyl@163.com (Y.Z.); 17696110015@163.com (H.J.); 17786451251@163.com (Q.Z.); 13871324158@163.com (Y.H.); 12683@whpu.edu.cn (L.Y.); xiaojjrr@126.com (J.X.); gssong2018@163.com (G.S.)
[2] School of Mathematics and Computer Science, Wuhan Polytechnic University, Wuhan 430023, China; wpnb20030916@163.com
[3] The Genetic Engineering International Cooperation Base of Chinese Ministry of Science and Technology, Key Laboratory of Molecular Biophysics of Chinese Ministry of Education, College of Life Science and Technology, Huazhong University of Science and Technology, Wuhan 430074, China; wgl_d2022@hust.edu.cn
* Correspondence: 12739@whpu.edu.cn (M.T.); yinli2021@hust.edu.cn (Y.L.)
† These authors contributed equally to this work.

**Supplementary Figure S1**. Identification of duplicated SbGRF gene pairs with the MCscanX software.

**Supplementary Figure S2**. Alignment of the full-length protein sequences of SbGRFs.

**Supplementary Figure S3**. The similarity matrix between SbGRF proteins.

**Supplementary Figure S4**. The gene expression patterns of OsGRFs and the evaluation on the expression similarities between OsGRFs.

**Supplementary Figure S5**. Construction of the co-expression networks with the Della internode RNA-seq data.

**Supplementary Table S1**. WGCNA identified co-expression networks of gene expression in the Della internodes. (in a separate EXCEL file)

**Supplementary Table S2**. The GO functional terms enriched in the SbGRF-containing modules of the Della co-expression networks. (in a separate EXCEL file)

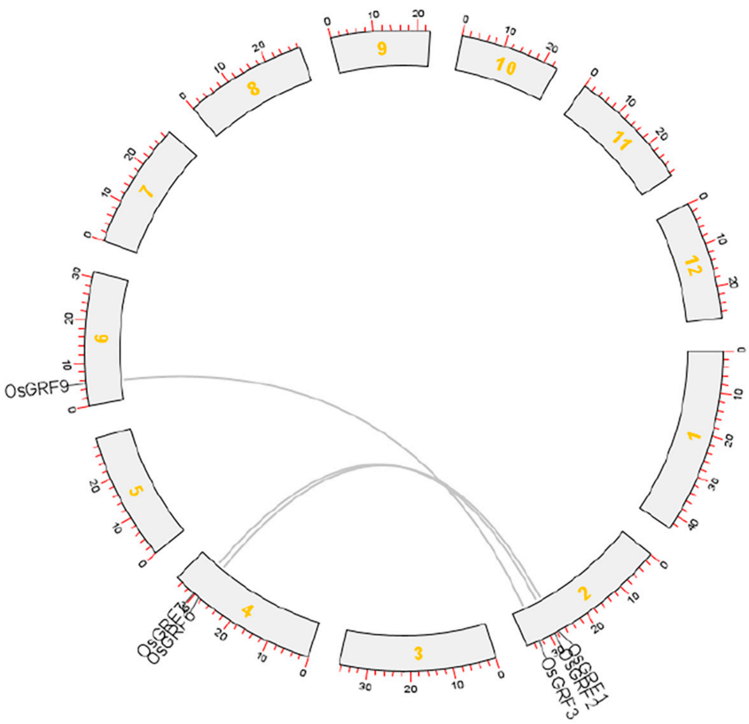


**Supplementary Figure S1**. Identification of duplicated *SbGRF* gene pairs with the MCscanX software. Default parameters were used for the analysis. The identified *SbGRF* pairs were lined with each other.

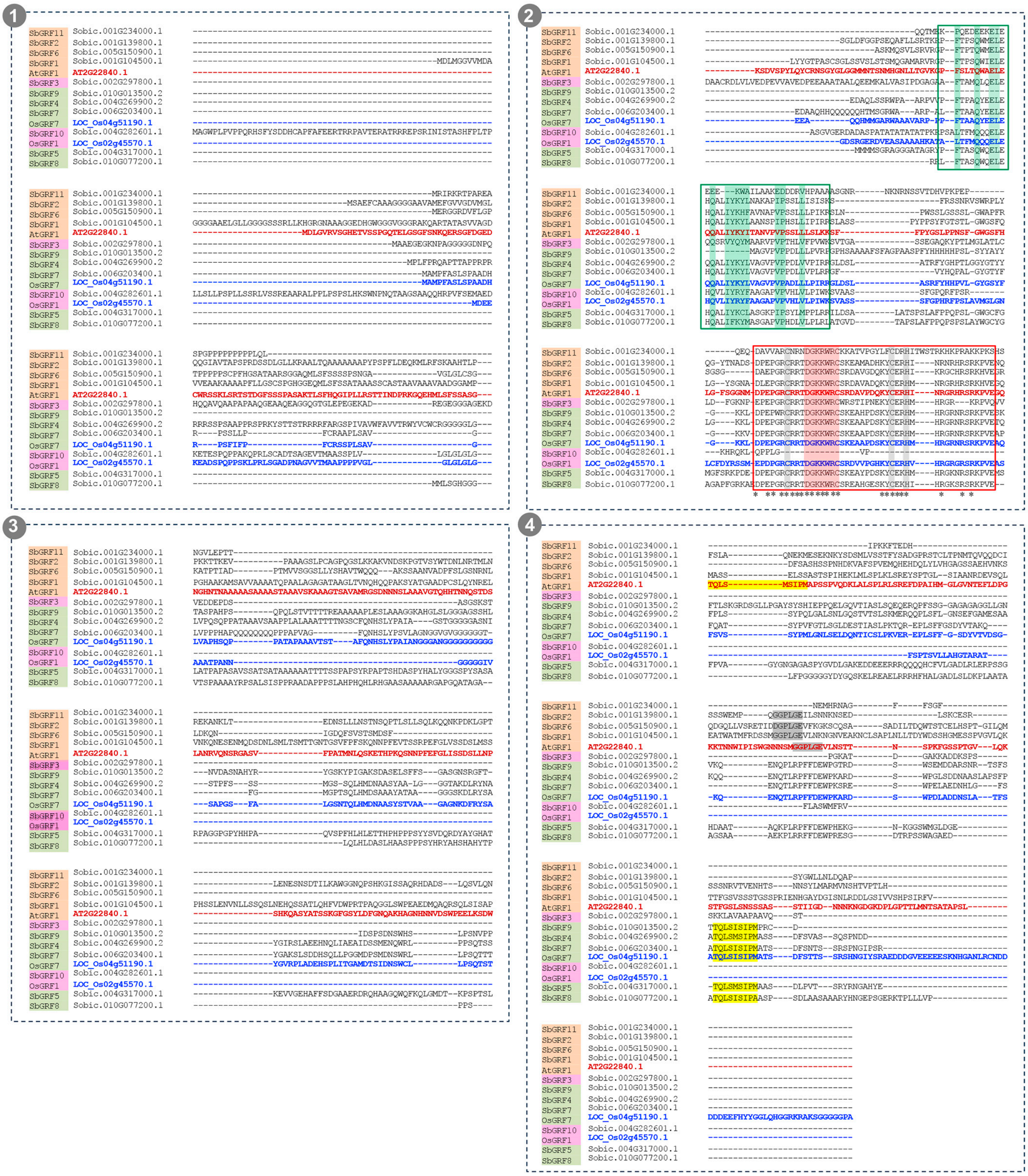

**Supplementary Figure S2**. Alignment of the full-length protein sequences of SbGRFs. The SbGRFs were aligned together with representative AtGRF and OsGRF proteins (AtGRF1, OsGRF1 and 7, respectively). The AtGRF and OsGRF sequences are indicated in red and blue fonts, respectively. Because the full-length protein sequences are long, the sequence alignment is split into four panels (labeled in numbers). The phylogenetic clades of GRF proteins are indicated in background colors (green, light orange, and pink to representing clades A, B, and C, respectively). On the panel 2, the QLQ and WRC domains are highlighted in green and red boxes, while on the panel 4, the TQL and GGPL motifs are indicated in yellow and grey backgrounds, respectively. These sequences were aligned with the MUSCLES method.

| | | OsGRF3 LOC_Os02g53690.1 | SbGRF5 Sobic.004G317000.1 | OsGRF9 LOC_Os06g10310.1 | SbGRF8 Sobic.010G077200.1 | OsGRF2 LOC_Os02g47280.1 | SbGRF4 Sobic.004G269900.2 | OsGRF7 LOC_Os04g51190.1 | SbGRF7 Sobic.006G203400.1 | OsGRF8 LOC_Os06g02560.1 | SbGRF9 Sobic.010G013500.2 | SbGRF6 Sobic005G150900.1 | OsGRF11 LOC_Os11g35030.1 | OsGRF12 LOC_Os12g29980.1 | OsGRF5 LOC_Os03g51970.1 | SbGRF1 Sobic001G104500.1 | OsGRF4 LOC_Os03g47140.1 | SbGRF2 Sobic001G139800.1 | SbGRF11 Sobic.001G234000.1 | OsGRF13 LOC_Os10g30880.1 | OsGRF1 LOC_Os02g45570.1 | SbGRF10 Sobic.004G282601.1 | OsGRF6 LOC_Os04g48510.1 | OsGRF10 LOC_Os07g28430.1 | SbGRF3 Sobic.002G297800.1 |
|---|---|---|---|---|---|---|---|---|---|---|---|---|---|---|---|---|---|---|---|---|---|---|---|---|---|
| OsGRF3 | LOC_Os02g53690.1 | 100 | 73 | 55 | 55 | 37 | 39 | 39 | 41 | 37 | 37 | 35 | 37 | 35 | 36 | 35 | 27 | 29 | 20 | 19 | 44 | 35 | 40 | 35 | 36 |
| SbGRF5 | Sobic.004G317000.1 | 73 | 100 | 50 | 51 | 37 | 41 | 41 | 42 | 39 | 38 | 35 | 35 | 38 | 38 | 37 | 27 | 28 | 20 | 22 | 44 | 32 | 38 | 33 | 34 |
| OsGRF9 | LOC_Os06g10310.1 | 55 | 50 | 100 | 61 | 42 | 39 | 41 | 45 | 45 | 41 | 41 | 40 | 38 | 38 | 36 | 30 | 30 | 18 | 21 | 49 | 39 | 48 | 39 | 39 |
| SbGRF8 | Sobic.010G077200.1 | 55 | 51 | 61 | 100 | 38 | 39 | 40 | 42 | 41 | 38 | 37 | 38 | 36 | 36 | 37 | 30 | 28 | 17 | 20 | 47 | 36 | 43 | 39 | 40 |
| OsGRF2 | LOC_Os02g47280.1 | 37 | 37 | 42 | 38 | 100 | 69 | 67 | 61 | 45 | 46 | 35 | 34 | 33 | 41 | 33 | 31 | 31 | 22 | 23 | 45 | 36 | 40 | 37 | 36 |
| SbGRF4 | Sobic.004G269900.2 | 39 | 41 | 39 | 39 | 69 | 100 | 63 | 61 | 42 | 45 | 37 | 34 | 34 | 41 | 33 | 31 | 32 | 19 | 21 | 42 | 35 | 38 | 36 | 35 |
| OsGRF7 | LOC_Os04g51190.1 | 39 | 41 | 41 | 40 | 67 | 63 | 100 | 74 | 46 | 48 | 38 | 40 | 35 | 40 | 37 | 32 | 33 | 20 | 22 | 45 | 39 | 41 | 36 | 34 |
| SbGRF7 | Sobic.006G203400.1 | 41 | 42 | 45 | 42 | 61 | 61 | 74 | 100 | 47 | 49 | 40 | 38 | 34 | 39 | 37 | 29 | 30 | 20 | 21 | 44 | 38 | 40 | 34 | 34 |
| OsGRF8 | LOC_Os06g02560.1 | 37 | 39 | 45 | 41 | 45 | 42 | 46 | 47 | 100 | 82 | 39 | 42 | 33 | 35 | 34 | 30 | 31 | 22 | 19 | 42 | 33 | 41 | 36 | 35 |
| SbGRF9 | Sobic.010G013500.2 | 37 | 38 | 41 | 38 | 46 | 45 | 48 | 49 | 82 | 100 | 33 | 35 | 31 | 33 | 32 | 26 | 26 | 27 | 21 | 41 | 28 | 39 | 35 | 36 |
| SbGRF6 | Sobic005G150900.1 | 35 | 35 | 41 | 37 | 35 | 37 | 38 | 40 | 39 | 33 | 100 | 63 | 48 | 44 | 41 | 36 | 34 | 19 | 21 | 51 | 40 | 48 | 36 | 33 |
| OsGRF11 | LOC_Os11g35030.1 | 37 | 35 | 40 | 38 | 34 | 34 | 40 | 38 | 42 | 35 | 63 | 100 | 47 | 43 | 42 | 35 | 34 | 18 | 20 | 44 | 33 | 42 | 35 | 30 |
| OsGRF12 | LOC_Os12g29980.1 | 35 | 38 | 38 | 36 | 33 | 34 | 35 | 34 | 33 | 31 | 48 | 47 | 100 | 47 | 42 | 38 | 36 | 17 | 20 | 44 | 34 | 43 | 32 | 30 |
| OsGRF5 | LOC_Os03g51970.1 | 36 | 38 | 38 | 36 | 41 | 41 | 40 | 39 | 35 | 33 | 44 | 43 | 47 | 100 | 82 | 38 | 39 | 18 | 22 | 47 | 37 | 42 | 37 | 34 |
| SbGRF1 | Sobic001G104500.1 | 35 | 37 | 36 | 37 | 33 | 33 | 37 | 37 | 34 | 32 | 41 | 42 | 42 | 82 | 100 | 36 | 37 | 17 | 18 | 39 | 28 | 39 | 34 | 30 |
| OsGRF4 | LOC_Os03g47140.1 | 27 | 27 | 30 | 30 | 31 | 31 | 32 | 29 | 30 | 26 | 36 | 35 | 38 | 38 | 36 | 100 | 71 | 20 | 21 | 42 | 32 | 36 | 30 | 29 |
| SbGRF2 | Sobic001G139800.1 | 29 | 28 | 30 | 28 | 31 | 32 | 33 | 30 | 31 | 26 | 34 | 34 | 36 | 39 | 37 | 71 | 100 | 22 | 23 | 39 | 29 | 35 | 30 | 29 |
| SbGRF11 | Sobic.001G234000.1 | 20 | 20 | 18 | 17 | 22 | 19 | 20 | 20 | 22 | 27 | 19 | 18 | 17 | 18 | 17 | 20 | 22 | 100 | 38 | 20 | 5 | 19 | 18 | 19 |
| OsGRF13 | LOC_Os10g30880.1 | 19 | 22 | 21 | 20 | 23 | 21 | 22 | 21 | 19 | 21 | 21 | 20 | 20 | 22 | 18 | 21 | 23 | 38 | 100 | 20 | 6 | 19 | 14 | 14 |
| OsGRF1 | LOC_Os02g45570.1 | 44 | 44 | 49 | 47 | 45 | 42 | 45 | 44 | 42 | 41 | 51 | 44 | 44 | 47 | 39 | 42 | 39 | 20 | 20 | 100 | 67 | 73 | 40 | 43 |
| SbGRF10 | Sobic.004G282601.1 | 35 | 32 | 39 | 36 | 36 | 35 | 39 | 38 | 33 | 28 | 40 | 33 | 34 | 37 | 28 | 32 | 29 | 5 | 6 | 67 | 100 | 62 | 34 | 37 |
| OsGRF6 | LOC_Os04g48510.1 | 40 | 38 | 48 | 43 | 40 | 38 | 41 | 40 | 41 | 39 | 48 | 42 | 43 | 42 | 39 | 36 | 35 | 19 | 19 | 73 | 62 | 100 | 40 | 42 |
| OsGRF10 | LOC_Os07g28430.1 | 35 | 33 | 39 | 39 | 37 | 36 | 36 | 34 | 36 | 35 | 36 | 35 | 32 | 37 | 34 | 30 | 30 | 18 | 14 | 40 | 34 | 40 | 100 | 79 |
| SbGRF3 | Sobic.002G297800.1 | 36 | 34 | 39 | 40 | 36 | 35 | 34 | 34 | 35 | 36 | 33 | 30 | 30 | 34 | 30 | 29 | 29 | 19 | 14 | 43 | 37 | 42 | 79 | 100 |

**Supplementary Figure S3**. The similarity matrix between SbGRF proteins. The protein similarity matrix reflecting the sequence identity between each pair of OsGRFs and SbGRFs were calculated with the Clustal-Omega program in the EMBL website. OsGRFs and SbGRFs were aligned with the Clustal-Omega method. The sequence similarity percentages (ranging from 0% to 100%) are shown in the heatmap with blue and red indicating the lowest and highest sequence similarity percentage, respectively. OsGRFs and SbGRFs belong to the same phylogenetic clades (Figure 1) are indicated with black boxes, and the gene names of OsGRFs and SbGRFs are given in green and purple fonts, respectively.

A

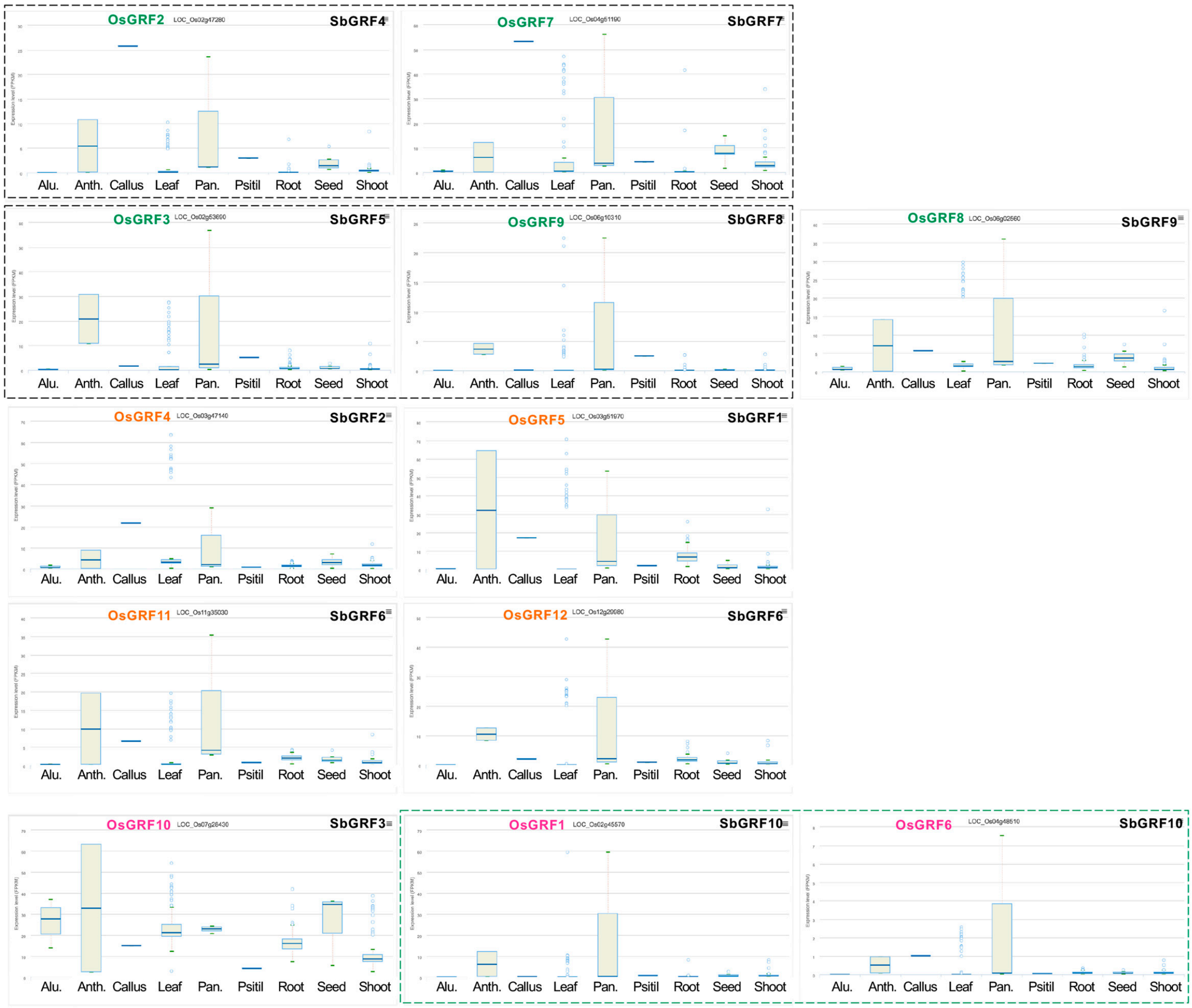


B

| Rice GRF | SbGRF | Alu. | Anth. | callus | leaf | panicle | seed | root | pistil | shoot | Note | |
|---|---|---|---|---|---|---|---|---|---|---|---|---|
| *OsGRF2* | *SbGRF4* | Very low | **Intermediate** | **High** | Very low | Low | Low | Very low | **Intermediate** | Very low | *GRF2* and *7* share a very similar expression pattern | *SbGRF4*'s expression pattern may be similar to *OsGRF2*'s |
| *OsGRF7* | *SbGRF7* | Very low | **Intermediate** | **High** | Very low | Low | **Intermediate** | Very low | **Intermediate** | Low | | |
| *OsGRF8* | *SbGRF9* | Very low | **Intermediate** | **Intermediate** | Low | Low | **Intermediate** | Low | Low | Low | The *GRF8* expression pattern is similar to those of *GRF2/7* | |
| *OsGRF3* | *SbGRF5* | Very low | **High** | Low | Very low | Low | Very low | Very low | **Intermediate** | Very low | *GRF3* and *9* share a very similar expression pattern | |
| *OsGRF9* | *SbGRF8* | Very low | **Intermediate** | Very low | Very low | Low | Very low | Very low | **Intermediate** | Very low | | *SbGRF8*'s expression pattern may be similar to *OsGRF9*'s |
| *OsGRF4* | *SbGRF2* | Very low | Low | **High** | Low | Low | Low | Very low | Very low | Very low | *GRF4* and *5* have different expression patterns | |
| *OsGRF5* | *SbGRF1* | Very low | **High** | **High** | Very low | Low | Very low | Low | Very low | Very low | | |
| *OsGRF11* | *SbGRF6* | Very low | **High** | **Intermediate** | Very low | Low | Low | Low | Very low | Low | *GRF11* and *12* share a very similar expression pattern | |
| *OsGRF12* | -- | Very low | **High** | Low | Very low | Low | Very low | Low | Very low | Very low | | |
| *OsGRF10* | *SbGRF3* | **High** | **High** | **Intermediate** | **High** | **High** | **High** | **Intermediate** | Low | Low | *GRF10* has a different expression pattern compared to *GRF1* and *6*. | *SbGRF3*'s expression pattern may be similar to *OsGRF10*'s |
| *OsGRF1* | *SbGRF10* | Very low | Low | Very low | Very low | Low | Very low | Very low | Very low | Very low | *GRF1* and *6* share a similar expression pattern except for the callus tissue. | *SbGRF10*'s expression pattern may be similar to *OsGRF1*'s |
| *OsGRF6* | -- | Very low | Low | **Intermediate** | Very low | Low | Very low | Very low | Very low | Very low | | |

**Supplementary Figure S4**. The gene expression patterns of *OsGRF*s (**A**) and the evaluation on the expression similarities between *OsGRF*s (**B**). **(A)** The gene expression patterns of *OsGRF*s across multiple tissues and stages collected from several studies were obtained from the IC4R rice database. The expression profiles of *OsGRF*s are sorted and plotted according to tissues and laid out based on their phylogenetic clades, which are indicated with the font color of gene names (green, orange and pink meaning clades A, B, and C, respectively). The orthologous *SbGRF*s are labeled in the corresponding expression plot. **(B)** Evaluation on the expression similarities between *OsGRF*s. The expression levels of *OsGRF*s were evaluated as “very low”, “low”, “intermediate”, and “high” to reflect the relative expression level of a given tissue type for each *OsGRF*. This evaluation is to compare and determine the tissue expression preference rather than to directly compare the absolute expression values between *OsGRF* genes. The background colors indicate the phylogenetic clades of *GRF*s, with the leaf, panicle, seed and root tissues indicated in red to highlight the four tissue types comparable with the expression of *SbGRF*s. Tissue abbreviations provided as follow: “Alu.”, aleurone; “Anth.”, anther; “Pan.”, panicle.

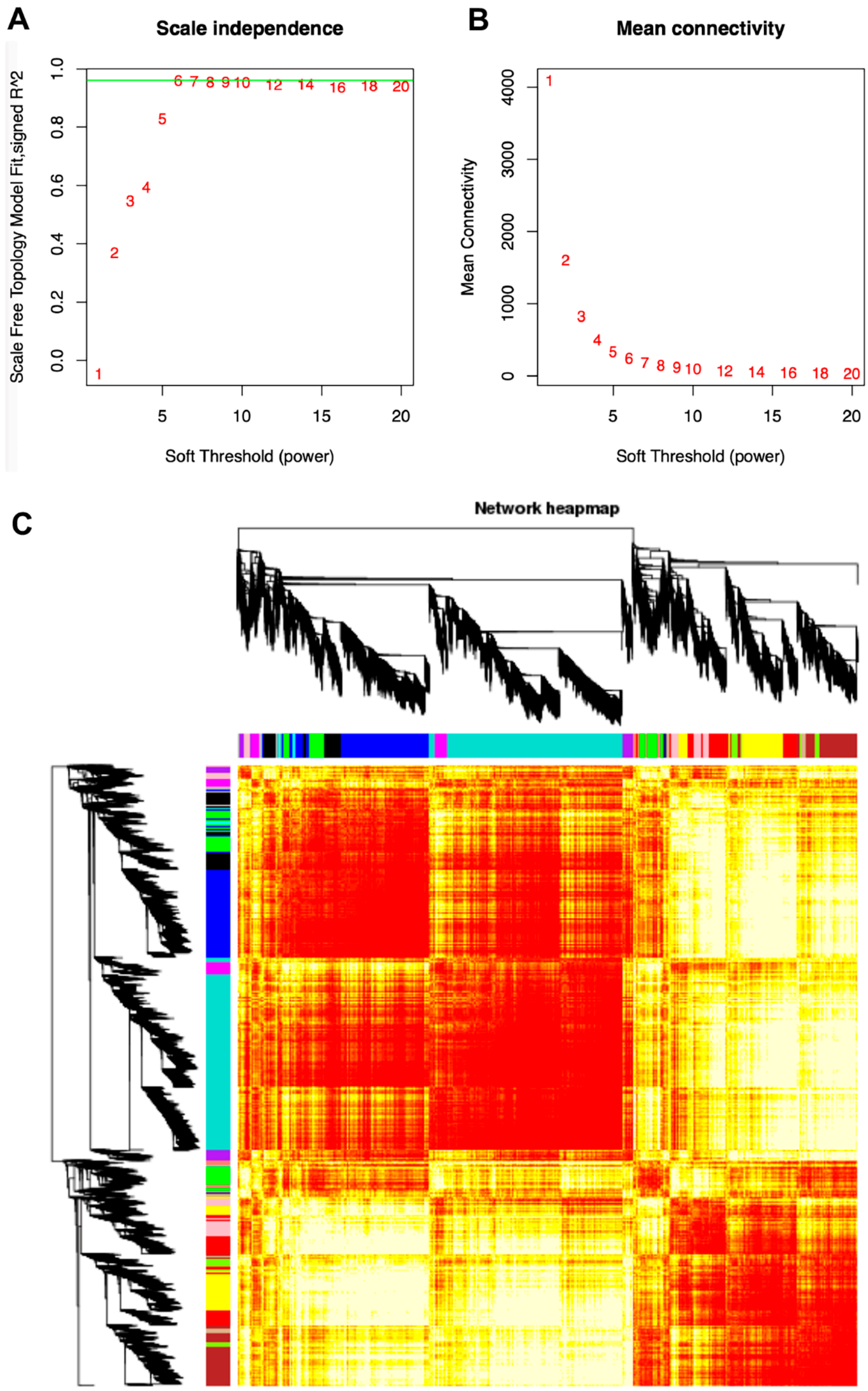


**Supplementary Figure S5**. Construction of the co-expression networks with the Della internode RNA-seq data. (**A** and **B**) Selection of soft threshold (power) for the construction of weighted gene co-expression network. After raising the co-expression network to a power threshold, the network topology is approximately scale-free. **(C)** the network heatmap to show that the genes used for co-expression network construction were separated into 15 co-expression modules with the gene dendrogram. The central heatmap indicates the levels of gene topology overlap (white indicates the levels of gene topology overlap are low, while red indicates high levels of gene topology overlap).
